# Physical Signs of the Chest

**Published:** 1872-04

**Authors:** W. L. Lipscomb

**Affiliations:** Columbus, Miss.


### PHYSICAL SIGNS OF THE CHEST.

BY W. L. LIPSCOMB, M. D., OF COLUMBUS, MISS.

(Read before the Columbus and Lowndes County (Miss.) Medical Society, March 12th, 1872.)

From the time the subject of diagnosis by physical signs was introduced in Vienna by Auenbrugger in the year 1761, and its further and more lucid development by those distinguished Frenchmen, Corvisart, Piorry, and Laennec, this department of medicine has received particular and enthusiastic attention, and its advocates have claimed for it the most definite, certain and advantageous results.

None can deny the immediate percussion of the walls of the chest by Auenbrugger, the use of the Pleximeter by Piorry, and the discovery and application of Auscultation by means of the stethoscope, by Laennec, constitute remarkable steps in the advancement of medical science. But it is equally true, that the advantages of this progress have been advocated and pursued with a recklessness and *abandon* detrimental to truth and to the welfare and existence of thousands of victims whose lives were sacrificed upon the altars of physical exploration. This was brought about by a neglect of those important vital symptoms and states of the constitution so necessary in arriving at a correct diagnosis and a magnification of the uncertain, indistinct, and confused evidences which the ear of a too anxious, enthusiastic discoverer or disciple could elicit from the different organs of the body in health and disease.

Modern medical science, with calm and unprejudiced skill, is endeavoring to separate the good and the true from that which is fanciful and injurious; and already sign after sign, and theory after theory, on careful trial and investigation, have been laid aside, until whole books have been reduced to pages, and a complicated science, which required the philosophic knowledge of Acoustics and the practiced ear of a musician has been reduced to a few fundamental rules, and within the scope of the dull ear of any student who seeks the acquisition.

The percussion theories of Piorry, drawn out until the brain and liver, and spleen, and heart, each gave forth their special sounds—the pathognomonic pectoriloquy and ægophony of Laennec—the consonating sounds of Skoda—have all failed to stand the *experimentum crucis*, and are to-day known to be the mistakes of honest but too hasty explorers in the new field of medical research.

In fact, signs as specially indicative of certain diseases, have all been abandoned by intelligent modern science. The indications to ear, or the physical signs of healthy organs, have been found to vary from the undeveloped condition of childhood, all along every state of these organs, through youth and manhood, to old age. No two portions of the same organ, on account of relative position or accidental circumstances, give the same sound, and even the different halves of the lungs do not correspond in their physical indications. And when disease comes in, and with its agencies of air, and serum, and blood, and mucous, and pus, produces a series of changes that extend from the derangement of a single air-cell to the involvment of a whole organ—from the inflated aerial distention of emphysema to the solid hepatization of Pneumonia—from the minutest bubble that crepetates, to the pints of fluid that obstruct all sound—well may modern science have concluded it was folly and madness to enter this labyrinth with but a single sound—with only an ear for our guide.

To-day, the physical signs of the chest occupy a place alongside of the indications of the pulse, the temperature of the skin, the expression of the face, the weight of the body, the character of the expectoration, the nature and seat of the pain—and, like them, are subjected to a rigid scrutiny, and to the same process of analytical and synthetical logic.

Do not suppose, Mr. President, that your essayist is prepared to eschew the important discoveries and undervalue the great advantages of physical signs as a means of diagnosis—or that he will deny that a certain degree of accuracy has been attained, by means of which the condition of the organs of the body may be determined.

We are only asserting that auscultation and percussion, separate and apart from other means of diagnosis, have not revealed signs pathognomonic of special diseases, by which they can be accurately determined.

Laennec, dumb and blind-folded, and with no taxis but the end of his stethoscope, would be lost amid the kindred bronchophonies of pulmonary infiltration and pneumonic hepatization.

Piorry might percuss his pleximeter in twain and not discover whether the *dullness* beneath it belonged to a pneumonia, consumption or pleurisy—and even Cammann, with a tube in each ear, would fail to distinguish the metalic tinkles of a tuberculous cavity and hollow pneumothorax.

The boasted crepitant rales of pneumonia are found associated with the pulmonary engorgement of fevers—oftentimes cannot be distinguished from the mucous rales of an acute bronchitis—and are not present to a sufficient extent to locate a lobular pneumonia.

What then has been taught with sufficient accuracy to justify us in undertaking the labor of acquainting ourselves with the signs and facts which this branch of science afford ? We will endeavor briefly to state :

1st. Percussion has taught us that the lungs yield *three* well marked, distinct sounds indicated by the terms :—1st, *clear ;* 2nd, *dull ;* 3d, *tympanitic.*

The *clear sound* is produced by the unhindered vibrations which are emitted from the air contained in the vesicles of healthy lungs, and is the standard from which disease is estimated.

This sound can be most readily learned by percussion of the healthy chest in the region below the clavicle and above the nipple.

*Dullness* denotes the absence of air, and indicates consolidation of the lung or the presence of something which checks the normal vibrations, and represents a class of diseases during the progress of which this consolidation or obstruction may happen—such as Phthisis, Pneumonia and Pleurisy. It may be learned by percussion of the thigh, or any other solid part of the body.

A tympanitic sound indicates an excess of air—above that contained in the small vesicles of the lungs, and is expression of emphysema, or pneumothorax, or a pulmonary cavity. It may be learned by striking the walls of the abdomen when distended by air in the intestines. Modifications of tympanitic resonance may be produced by plumping the cheek, or striking the hands with the palms clasped together upon the knee.

Auscultation reveals two general characters of sounds: First, the modifications of the normal respiration. Second, non-adventitious sounds—peculiar to diseased conditions. Under the first head are included the *vesicular murmur*, and *bronchial respirations*. The *vesicular murmur* is the natural sound of the healthy lung, and is produced by the entrance of air into the pulmonary tissue, and its consequent distention. It may be compared to the slightly audible breathing heard at a little distance from a person in a quiet, deep sleep—to a sound produced by a gentle breeze among the branches and leaves of the trees—and it may be imitated by the mouth, when placed in proper position and pronouncing the syllable *ve* or *vu*.

*Bronchial respiration* is the sound produced by the motion of air in the larger and smaller bronchial tubes, and is an evidence of disease—this sound being obscured in health by the vesicular murmur. The diseases to which it is peculiar are those in which the pulmonary tissue has become hardened or condensed—such as pneumonia, pleuritic effusion, pulmonary apoplexy, and tubucular infiltration. This sound may be imitated bp blowing in a tube, or by pronouncing with the mouth a gutteral *cho*, or *chu*.

The second general division of new and adventitious sounds is sub-divided as follows: *rales*, *or ronchi*, and *friction sounds*.

The *rales* are sounds which are generated in the air-tubes by the passage of air through them, when contracted, or when containing fluid.

When the air-tubes are contracted by thick, tenacious fluids, which the passage of the air cannot break up, *dry rales* are produced—which resemble in sound the cracking of leather or frozen snow. When they contain a thin fluid which the passage of air breaks into bubbles, *moist rales* are produced. These conditions mostly transpire in the larger and smaller bronchial tubes, and indicate disease in these localities.

Again—another *rale* is produced by the presence of fluid in the air-cell, which is called the *crepitant rale.* It resembles the noise occasioned by throwing salt on the fire, or by rubbing the hair between the fingers. This *rale* is heard only during inspiration, and is the nearest of all physical signs to a pathognomonic symptom of disease—viz : pneumonia.

*Friction sounds* are produced by the rubbing together of the roughened surfaces of an inflamed pleura—generally audible upon but one side and at the lower portion of the chest. The well-marked friction sound may be imitated by placing the palm of the left hand over the ear, with firm pressure, and moving slowly over the dorsal surface the end of the finger of the right hand. This sound, when present, indicates pleurisy ; but it is comparatively valueless, because in about 90 per cent of the cases it is entirely absent.

Your essayist has, he trusts, carefully collected for your consideration all the sounds evinced by auscultatory exploration, as recognized by the highest authorities of the present day, and concerning which all are agreed. It is true, each author might add some additional sign, which he regards clear and well established—but from the fact that it has failed to secure the sanction of all, we have omitted it.

The practitioner, in the use of these signs, must, of course, take them in connection with all the other symptoms of disease, and the physical conditions evinced by pathology and morbid anatomy. Especial regard will be paid to their location in the chest—and he will soon begin to generalize, perhaps not inaccurately, as follows : An extensive diffusion of rales over a large portion of the posterior surface of both lungs, may be set down as bronchitis in some form or stage. Dullness on percussion, crepitation and bronchial respiration over the inferior lobe of either lung, but especially of the right, and most easily distinguished particularly below the scapula, indicates pneumonia ; while the upper lobe of either lung, but more especially the left, present an ominous gatherer for the collection of all the signs—and any one of them significant of consumption.

The art of auscultation and percussion has been considerably modified by modern science. The fingers of the left hand are found to be equally as appropriate as the pleximeter to receive the percussive strokes, while the fingers of the right hand make just as efficient a hammer as the instrument used for that purpose. Immediate auscultation with the ear has almost superceded the stethoscope—that instrument maintaining its superiority only in the examination of particular, inaccessible portions of the chest, and on account of delicacy in the examination of females.*

The most approved position for the patient in examination of the anterior surface, is standing with the back against a wall, the shoulders moderately back and firmly supported—or sitting in a high-back chair in same position. In examining the back, the body should be inclined forward and the arms folded, while sitting upon a stool or chair. In exploring the lateral surfaces, the hands should be clasped above the head.

If the patient is confined to bed, the chest in front may be examined in the recumbent position, and afterwards he may be raised and supported in sitting posture while the examination is made behind and laterally.

Your essayist feels indisposed to close this paper without paying a tribute to the genius and merit of the great discoverer of auscultation, who, though he may have failed to furnish us characteristic signs of each disase, has contributed more than any other man to the establishment of the distinctive pathology and morbid anatomy of diseases of the chest. We recommend his work on "Diseases of the Chest," so far as physical diagnosis is concerned, as superior to any since written. Its style has that simple, charming expression of thought and detail of fact peculiar to the French, and not dissimilar to our own brilliant Dewees and Eberle, and is in striking contrast to the dull, verbose, stilted and pedantic style of modern American authors.

The German Skoda is another very interesting author, and while possibly more imaginary in his views than Laennec, himself, still he ranks among the most successful writers, who under-

---

*The Pleximeter and Stethoscope are not materially changed from their original construction, and no great superiority claimed for any recent manufacture.

took to corect the errors into which Laennec's zeal as a discoverer had led him.

Stokes, Gerhand, Barthez & Rolliet, Flint & DeCosta are good books of reference—especially as compilations of the advances made by modern science in the simplification of physical diagnosis, and as corrections of the mistakes of the early discoverers and theorists in this field of medicine.

